# Adaptive capabilities and fitness consequences associated with pollution exposure in fish

**DOI:** 10.1098/rstb.2016.0042

**Published:** 2017-01-19

**Authors:** Patrick B. Hamilton, Gregor Rolshausen, Tamsyn M. Uren Webster, Charles R. Tyler

**Affiliations:** 1 Biosciences, College of Life and Environmental Sciences, University of Exeter, Exeter EX4 4QD, UK; 2 Senckenberg Biodiversity and Climate Research Centre (BiK-F), Senckenberganlage 25, 60325 Frankfurt am Main, Germany; 3 Department of Biosciences, Swansea University, Wallace Building, Swansea SA2 8PP, UK

**Keywords:** adaptation, fish, chemical pollution, genetic adaptation, single-nucleotide polymorphism, tolerance

## Abstract

Many fish populations are exposed to harmful levels of chemical pollution and selection pressures associated with these exposures have led to the evolution of tolerance. Our understanding of the physiological basis for these adaptations is limited, but they are likely to include processes involved with the absorption, distribution, metabolism and/or excretion of the target chemical. Other potential adaptive mechanisms include enhancements in antioxidant responses, an increased capacity for DNA and/or tissue repair and alterations to the life cycle of fish that enable earlier reproduction. Analysis of single-nucleotide polymorphism frequencies has shown that tolerance to hydrocarbon pollutants in both marine and estuarine fish species involves alteration in the expression of the xenobiotic metabolism enzyme CYP1A. In this review, we present novel data showing also that variants of the CYP1A gene have been under selection in guppies living in Trinidadian rivers heavily polluted with crude oil. Potential costs associated with these adaptations could reduce fitness in unpolluted water conditions. Integrating knowledge of local adaptation to pollution is an important future consideration in conservation practices such as for successful restocking, and improving connectivity within river systems.

This article is part of the themed issue ‘Human influences on evolution, and the ecological and societal consequences’.

## 1. Introduction

Pollution is a major threat to populations of wild fish. Habitats under chronic exposure to pollutants often suffer reduced species richness and loss of community integrity [1–3]. These changes can substantially impact aquatic ecosystems and, thus, the services they provide [4]. At the same time, rapid adaptive evolution of tolerance to pollutants has been shown to occur in several fish species [5–7]. This could limit, or severely modify ecosystem impacts. The genetic basis for evolved tolerance is now understood in a handful of species for a few chemicals. These include: Atlantic killifish (also known as mummichog, *Fundulus heteroclitus*) exposed to polycyclic aromatic hydrocarbons (PAHs) [5,8,9], Atlantic tomcod (*Microgadus tomcod*) exposed to polychlorinated biphenyls (PCBs) and dioxin-like substances [10,11], yellow perch (*Perca flavescens*) exposed to cadmium [12] and eels (*Anguilla anguilla* and *Anguilla rostrata*) exposed to metals and organic pollutants [13]. However, in most cases, adaptive mechanisms and their fitness consequences are unknown.

Understanding the ability of fish to adapt to chemical pollution is important for informing how evolutionary dynamics resulting from anthropogenic disturbance may affect conservation and management decisions [14]. This is because pollution often occurs on contemporary timescales, so persistence of the affected populations may require equally rapid adaptive responses from standing genetic variation [15].

In this review, we consider the physiological mechanisms involved in genetic adaptation to chemical pollutants. We define adaptation as genetic change at a population level that results from evolution, through selection on traits that confer a survival or reproductive advantage. We do not consider phenotypic responses resulting from exposure, which could also enhance tolerance (phenotypic plasticity) [16]. Local adaptation is likely to occur predominantly from selection on standing genetic variation such as single-nucleotide polymorphisms (SNPs) but may also involve novel mutations. Established physiological mechanisms involved in evolved tolerance in fish have been reviewed by Van Veld & Nacci [17] and by Di Giulio & Clark [9]. Here, we build on these reviews to also consider hypothetical adaptations and their potential impacts on fitness in unpolluted water conditions.

Gene expression responses to xenobiotic exposure, and known mechanisms involved in tolerance are useful for hypothesizing adaptive mechanisms in fish [5,11]. Another source of information is the rapidly increasing knowledge of the influence of variants in human genes on the outcome of exposures to xenobiotics, such as pharmaceuticals and PAHs (e.g. from smoking). A recent study identified local adaptation in human populations exposed to arsenic in drinking water [18]; but in most cases these human variants have been linked to individual susceptibility, not to population-wide local adaptation. Fish and humans have many common physiological mechanisms and targets for toxins; approximately 70% of human genes have at least one obvious zebrafish (*Danio rerio*) orthologue [19]. However, in contrast to humans, fish often absorb pollutants from the water and experience higher exposure concentrations generally. Major drivers for the identification of such mechanisms are to establish (i) whether local adaptation to pollution will have population-level implications for existence in the wider environment (i.e. do adaptations to pollutants entail fitness costs when these pollutants are absent?); (ii) whether fish adapted to one pollutant are better, or less, able to cope with other pollutants or stressors in a changing environment; and (iii) considerations for restocking in conservation practices.

In the near future, our understanding of adaptive mechanisms is likely to advance through the identification of loci with signatures of selection from genomic comparisons between populations [20]. The function of genes in, or close to, these genomic regions can suggest traits that enhance lifetime reproductive fitness (i.e. survival × reproduction) in the polluted environment [5,13]. However, quantifying the influence of adaptive alleles on survival and/or reproductive output also requires knowledge of the number of generations of exposure, the demographic history of the population and the historical effective population size. In most cases, these will not be known. Reciprocal transplant experiments can also be used to quantify fitness differences. Obtaining meaningful data here may require assessments of reproductive output over a lifetime of exposure. Furthermore, F_2_ fish are required to separate the direct effects of exposure from genetic influences [9].

The impact of enhanced relative fitness (fitness of a resident compared to an immigrant) on population growth and persistence is complicated by a number of factors. For instance, absolute fitness (lifetime reproductive success) could be impacted despite considerable adaptation to chemical exposure, if adaptive traits have fitness costs and/or do not fully protect from the adverse effects of exposure [21]. Population growth rates of pollution-adapted populations at contaminated sites could therefore be considerably reduced. Populations could even decline to extinction while adaptation is occurring. Absolute fitness has greater ecological and societal relevance, as it potentially alters both the age/size distributions and the densities of fish populations. The influence of adaptations on population sizes will be further complicated by the effects of density-dependent growth and survival which play major roles in the regulation of population sizes in some fish species [22]. Furthermore, population sizes will be strongly influenced by the effects of pollution on competitors, predators and prey [23].

## 2. Contamination patterns and adaptation in marine and freshwater habitats

The pollution landscape is highly variable across freshwater and marine systems. In some cases, there are extremely high levels of pollution with a limited geographical distribution, for example, resulting from metal pollution from mining activities [24], discharges of pharmaceuticals/pesticides from manufacturing plants [25,26] and oil leaks from extraction sites [27]. Other contaminants can be widespread. Examples include effluents from waste water treatment works (WwTW) that occur in most lowland rivers within the UK [28], acid rain resulting from atmospheric emissions that falls over very wide geographical areas, notably in Northern Europe [29,30] and dichlorodiphenyltrichloroethane (DDT) metabolites from agricultural run-off that have been detected in Baltic Sea fish [31]. Local adaptation may be more widespread in freshwater habitats, due to limited dilution of contaminants and the presence of features (e.g. waterfalls, dams and weirs) that limit dispersal, and maladaptive gene flow. Equally, however, the small size of many freshwater habitats could make adaptation less likely because populations may go extinct before adaptations have time to develop, or due to the absence of the genetic variation required to adapt.

Where contamination is patchy and there are high levels of gene flow/migration between contaminated and unpolluted sites, selection within the contaminated sites could lead to increased tolerance at unpolluted sites. This will depend largely on whether these variants incur a cost in unpolluted water conditions. Given strong selective pressures, local adaptation can still occur despite high gene flow between unpolluted and polluted environments, as has been shown in *F. heteroclitus* from highly polluted estuarine habitats in the USA [5] and three-spined stickleback (*Gasterosteus aculeatus*) exposed to pulp mill effluents [32].

## 3. Physiological mechanisms for adaptations and their potential fitness costs

### (a) Adaptations to low oxygen

Nutrient-rich pollution that may be derived from fertilizers and poorly treated sewage has led to substantial problems with eutrophication as well as hypoxia and anoxia in both marine and freshwater habitats [33,34], often resulting in a shift towards more tolerant species [35,36]. Fish adopt a variety of physiological strategies to cope with hypoxia. These include alterations in respiration rate, alterations in behaviour (such as aquatic surface respiration) and even gill structure remodelling [37]. Reduced oxygen will likely create a strong selective pressure that could lead to the evolution of populations with increased hypoxia tolerance [36]. Many physiological (not evolutionary) responses to hypoxia in vertebrates are activated through the hypoxia-inducible factor (Hif) pathway and include altered blood cell production and vasodilation [38]. In some human populations that live at high altitude, polymorphisms of two genes that interact with the Hif pathway alter haemoglobin phenotypes and enable tolerance of environmental hypoxia [39]. Polymorphisms in globin genes play a role in local adaptation of populations to low available oxygen for some marine fish species. This includes populations of European hake (*Merluccius merluccius*) [40] that have adapted to different water temperatures [38]. Components of the Hif pathway and polymorphisms in globin genes therefore are candidates for genetic adaptation to pollution-driven hypoxia. Potential costs of such adaptations could be suboptimal oxygen use in oxygenated water, and metabolic costs in the higher production of haemoglobin.

### (b) Absorption, distribution, metabolism and excretion

Many of the potential adaptations to pollution involve physiological processes that regulate the level of internal chemical exposure, such as absorption, distribution, metabolism and/or excretion (ADME) [17]. These processes together describe levels and kinetics of internal chemical exposure and clearance, and hence influence toxicity. In general, concentrations of the respective chemical (or its metabolites) in the plasma of exposed fish are good predictors of biological effects [41] but they can also show considerable intra-individual variation. As an example, individual plasma concentrations in fathead minnow (*Pimephales promelas*) exposed to a synthetic glucocorticoid differed by as much as eightfold, which may in part be attributed to high variability in absorption and excretion dynamics [41].

Almost nothing is known of adaptations that reduce the uptake of chemicals from the environment, but this may be an important adaptive mechanism. Evolutionary adaptations are likely to involve organs and tissues that are in direct contact with the chemical agent, such as gills, skin and gut epithelium as well as the outer layer (chorion) of eggs. In humans, a range factors are known to influence uptake of chemicals from the gut into blood circulation. These include mucus production [42] and polymorphisms in genes for transport proteins such as divalent metal transporter 1 (for metals) [43] and multidrug resistance protein 1 (MDR1; for pharmaceuticals) [44]. In humans, gut microbial community composition also influences xenobiotic metabolism [45] and can be influenced by host genotype [46]. Free-living microbial communities can reduce copper availability at contaminated sites [47]. In fish, there is evidence for the potential to adapt by limiting accumulation. As examples, least killifish (*Heterandria formosa*) selected for cadmium tolerance in the laboratory and wild populations of metal tolerant *P. flavescens* had improved abilities to limit accumulation [6,7]. The mechanisms involved in both species were not determined. Physiological acclimatization of fish to metals involves increased proliferation and hypertrophy of mucus-producing goblet cells; mucus chelates metals, reducing their availability [48]. MDR1, which pumps xenobiotics (such as toxins or drugs) back into the intestinal lumen, is a prime candidate for transport-related adaptation in fish [49]. The expression of this gene in *F. heteroclitus* from a contaminated site was shown to be two- to threefold higher than in fish from reference sites, but whether this resulted from adaptation is not known [50]. Therefore, genes that influence mucus production, gut transport and the gut microbiome could play important roles in the evolution of tolerance to pollutant exposure.

In fish, gills play important roles in gas exchange, ionic regulation, acid–base balance and nitrogenous waste excretion and are another major site of absorption of toxins [51]. Various environmental pollutants (e.g. metals, acid rain and PAHs) are known to affect gill morphology, and acute metal toxicity is known to disrupt ion homeostasis through inhibition of ATPases and carbonic anhydrase [52]. Evolutionary adaptations could involve altered regulation or specificity of gill transport proteins to reduce active uptake or increase transport away from gills. For example, the gills of metal tolerant wild brown trout (*Salmo trutta*) had altered the expression of genes related to metal transport (e.g. divalent metal transporter 1, cellular transporters of copper and copper-transporting ATPases). This suggests that metal homeostasis mechanisms are involved in limiting uptake of metals from the environment, or increasing clearance from the gill, although whether this is the result of genetic adaptation is not known [53]. These metal tolerant brown trout still accumulated metals in the gills and other tissues suggesting altered metal transport is not the only mechanism of tolerance [53].

Adaptations could potentially alter the internal distribution of contaminants in the body of the fish. Many substances are transported in the blood associated with proteins. For example, in vertebrates, steroid oestrogens are associated with sex hormone-binding globulin, and metals are associated with proteins such as glutathione, ferritin and metallothionein [54,55]. Altered quantities of such blood proteins could influence absorption by tissues. Several internal barriers could affect distribution by affecting chemical access to tissues. As with absorption, there is greater potential to modify internal distributions of substances that are actively transported across membranes such as metals and larger lipophilic molecules. For example, in mammals MDR1 in capillary endothelial cells of the blood–brain barrier pumps out xenobiotics and toxins, preventing access to the brain [56]. Adaptations involving this gene could therefore potentially restrict access of pollutants to the brains of exposed fish.

Sequestration of toxicants into body or cellular compartments where their toxic effects are mitigated is known to occur for various toxicants, but this has yet to be shown as a mechanism in local adaptation. Such sequestration systems allow for enhanced tolerance to a given toxicant, but it is likely that even with adaptations to increase the ability to sequester materials, continuous exposures will result in toxicity. As an example, in humans, prolonged lead exposure (that is sequestered into bones) eventually results in neurological effects [57]. In fish, sequestration of metals including copper, silver, cadmium, zinc, mercury and arsenic involves metallothioneins. These proteins bind harmful free metal ions within cells reducing their availability [58]. Other important sequestration molecules include ferritin, transferrin and glutathione. Metallothionein-bound metal ions can be metabolically detoxified or stored in metal-rich granules, particularly in the liver [59]. Upregulation in metallothionein genes has been observed for fish living in metal-contaminated rivers [53,60], but as yet it is not known whether this relates to adaptation. Lipophilic contaminants such as DDT metabolites, polybrominated diphenyl ethers and PCBs are incorporated into fats where they may be less harmful, at least until fats are subsequently metabolized [61]. Adaptations that involve sequestration could involve increased production of sequestration molecules, or increased affinity of sequestration molecules to specific pollutants.

Adaptations could involve altered metabolism of the pollutant. For organic pollutants, this is likely to involve phase I metabolism by cytochrome P450 enzymes and phase II enzymes such as glutathione S-transferases that play essential roles in the metabolism and are required for excretion through bile [62]. In humans, polymorphisms in genes for several enzymes (CYP1A1, CYP1B1, glutathione S-transferase Mu (GSTM)1, GSTM3) have been associated with altered susceptibility to DNA–PAH adduct formation and cancer [63], but more recent studies have indicated fewer convincing associations involving P450 genes from families 1 to 3 [64]. Some human populations have adapted to high arsenic content in the drinking water, which has been attributed to variants of the arsenic methyltransferase gene (*AS3MT*) [18]. In fish, variants in phases I and II metabolism genes will almost certainly modify response to a wide range of organic contaminants. Indeed CYP1A is refractory from induction in populations of both *F. heteroclitus* and *M. tomcod* which have adapted to exposure to both PAHs and PCBs/dioxin-like compounds [8,10,11]. In *F. heteroclitus*, the genetic polymorphisms responsible are two SNPs in the regulatory regions of the CYP1A gene [8], whereas in resistant *M. tomcod*, the aryl hydrocarbon receptor 2 (AHR2) variant has poor binding affinity to the dioxin-like compounds, limiting downstream CYP1A activation [11]. AHR2 variants may also be involved in toxicity tolerance in of some *F. heteroclitus* populations [65,66].

There are potential costs of adaptations related to ADME. The production of mucus may have energetic costs, could restrict uptake of nutrients and could also disrupt pathogen defence mechanisms. Presumably altered regulation of CYP1A impacts on the ability to detoxify other contaminants, and altered binding affinity of AHR2 to dioxin-like compounds may also impact binding of the endogenous ligand(s). Studies in humans and mice (*Mus musculus*) have shown that the AHR gene is important for immune system functioning [67,68], suggesting potential immune-related costs of these adaptations. Interestingly, *F. heteroclitus* populations resistant to PAH exposure differ in their immune function and susceptibility to disease (e.g. [69]) and are more sensitive to hypoxia and fluoranthene-mediated phototoxicity [70]. It has not yet been possible to relate these phenotypes to specific genetic variants in the population. Therefore, from the limited evidence in fish, and by extrapolation with studies in mammals, adaptations involving metabolism are likely to play a key role in adaptations of fish to a wide range of pollutants.

### (c) Target genes and disrupted processes

Target site polymorphisms may play an important role in resistance to pollutants with specific biological targets or where response is mediated through specific receptors. This has occurred in some insects resistant to pyrethroid insecticides [71]. In humans, polymorphisms in target genes alter response to pharmaceuticals [72]. It is therefore likely that such variation exists in fish. Many fish are exposed to pharmaceuticals through WwTW effluent discharges, although environmental concentrations are generally below those known to exert biological effects [73]. There are exceptions, such as the synthetic oestrogen ethinyloestradiol, a component of the human contraceptive pill, that feminizes male fish and reduces egg output at low concentrations [74]. The involvement of receptor polymorphisms in resistance is demonstrated by AHR2 polymorphisms in the tolerance of *M. tomcod* to PCBs [11].

For chemicals that disrupt specific biological processes, adaptations could involve genes that regulate these processes to compensate for the chemical impact. A very recent example of this comes from eels (*A. anguilla* and *A. rostrata*) inhabiting rivers polluted with various DDT metabolites, PCBs, metals and other anthropogenic pollutants. Laporte *et al.* [13] found that variants of genes involved in sterol regulation, transport processes and regulation of the digestive system played an important role in the survival of eels in polluted rivers. A single gene, ATP-binding cassette transporter (ABCG)5, was found to be under selection in both species. This transporter limits intestinal absorption and promotes biliary excretion of sterols. A range of other genes involved in sterol metabolism were also found to be important in each species. Laporte *et al*. [13] hypothesized that the observed selection related to differences in the ability to detoxify PCBs through the monooxygenase system, which is inhibited by steroid hormones. That study found that the majority of adaptation was caused by small shifts in allele frequency in many covarying loci, each with a small effect, rather than pronounced changes at a few loci with large effects. As biological/physiological traits often are commonly influenced by variation in multiple genes, adaptation involving multiple genes may be common [75].

Adults of both eel species (*A. anguilla* and *A. rostrata*) migrate to the Sargasso Sea to reproduce, and population-genetic studies suggest that *A. rostrata* comprises a single population [76]. Therefore, selection of genotypes in contaminated rivers could potentially lead to the evolution of tolerance of the species as a whole. However, this will depend on the success of eels from contaminated rivers migrating to the Sargasso Sea to reproduce, and whether the genotypes selected upon in polluted rivers incur a cost. Costs of adaptation involving genes that alter sterol metabolism could include effects on the production of steroid hormones such as oestrogen and testosterone, with implications for reproduction or blood cholesterol levels. In humans, both low and high blood cholesterol levels are associated with adverse health impacts and polymorphisms in ABCG transporters and are linked to hypercholesterolaemia and gallstone disease [77].

### (d) Oxidative stress and damage repair

A wide range of chemical contaminants including metals, pesticides and PAHs induce oxidative stress in exposed fish, and short-term responses in individuals include altered regulation of genes involved in oxidative stress response and DNA repair (e.g. [78]). Therefore, genetic adaptations at a population level that involve these processes have the potential to enhance tolerance to a wide range of pollutants. In humans, polymorphisms in genes involved in oxidative stress response (superoxide dismutases) and DNA repair are associated with altered susceptibility to the DNA–PAH adducts and various cancers [79,80]. Local adaptation of *Drosophila melanogaster* populations exposed to DNA-damaging ultraviolet light at high altitude involves polymorphisms in DNA-repair genes [81]. No such adaptations are known in fish, but by extrapolation, variants in such genes are likely to play a role in individual susceptibility and the evolution of tolerance of fish populations exposed to chemicals that induce DNA damage. Potential costs of evolutionary adaptations that enhance DNA repair could include slower DNA replication and reduced growth rate [82]. Moreover, enhanced ability to repair one type of DNA damage can increase other mutations [83], or could impact antibody production which relies on many of the same pathways [84]. It has also been argued that mutations that result in liver tumours could enhance survival in contaminated environments, as the tumours still maintain liver function yet have greater resistance to the toxic by-products of metabolism of various contaminants [17].

### (e) Metabolic costs of toxicity responses

Processes such as metabolism and excretion of the pollutant, the production of mucus and sequestration molecules, the repair of tissues and molecules and replacing damaged cells, will incur metabolic costs. Hence adaptations that involve these processes are likely to also be energetically costly. Experiments that selected for increased tolerance to cadmium in *H. formosa* found an associated 18% decrease in fecundity, due presumably to reallocation of energy resources away from reproduction and towards tolerance mechanisms [85]. Metabolic costs of tolerance could therefore have a major impact on the type of adaptations that develop. Where only a part of the habitat is polluted, or where exposure concentrations fluctuate, it may be advantageous to rapidly downregulate costly tolerance mechanisms when contamination concentrations are low to conserve resources. By contrast, in a uniformly polluted environment, an ability to turn off tolerance responses will offer no selective advantage, potentially leading to the evolution of adaptations that would be costly in unpolluted water. Such adaptations could include elevated baseline expression of genes involved in detoxification or sequestration.

### (f) Timing of reproduction/life cycle completion

Where chemical pollution results in high mortality and/or decreases fitness in later life, traits that allow for a faster than normal life cycle completion, i.e. fast growth and early reproduction, may be important targets for selection. Likewise, fish may adapt through early reproduction rather than through metabolically costly tolerance mechanisms that divert resources from growth and reproduction [86]. In humans, genes have been identified in which variants influence the age of onset of puberty. These include the follicle-stimulating hormone (FSH) receptor [87] and vestigial-like family member 3 (vgll3) [88] that also affects timing of puberty in Atlantic salmon (*Salmo salar*) [88]. There is some evidence that fish at contaminated sites have altered life cycles. For example, metal tolerant *F. heteroclitus* from a contaminated creek in the USA have been shown to reach maturity earlier than reference fish, but had shorter lifespans [17,89]. A similar finding has been reported for *P. flavescens* in metal-polluted lakes, where these fish tended to ‘live fast, die young’ [7]. As with all observations on wild fish, it is extremely difficult to separate the effects of exposure from adaptations. However, comparisons of SNP frequencies between these *P. flavescens* populations identified signatures of selection in components of the p53 pathway, which plays a fundamental role in controlling the cell cycle and is potentially linked to fast life cycle completion [12]. This situation is similar to selection through size-selective harvesting of cod (*Gadus morhua*) [90] and high predation of adult Trinidad guppies (*Poecilia reticulata*) [91] which have also led to earlier reproduction. There can be costs associated with early reproduction, including smaller eggs and reduced offspring survival, as has been shown to occur in *G. morhua* [90]. Therefore, while the benefits of adaptation would outweigh these costs in polluted environments, these adaptations could equally also reduce overall lifetime reproductive success and therefore negatively impact population growth rates.

Importantly, life cycle completion differs from other adaptations considered here as it has the potential to enhance lifetime reproductive success (in polluted water) irrespective of the mode of action of the pollutant. This may be important where the spatial distribution of contamination is complex, leading to different local selective pressures, and also where fish are simultaneously exposed to many different chemicals that exert adverse effects through different modes of action.

## 4. The Trinidad guppy (*Poecilia reticulata*) as a model to study the evolution of tolerance to chemical pollution

The limited current knowledge of the mechanisms for adaptation to chemical pollution and their fitness costs illustrates the need for more studies and more fish models to improve our understanding. In Trinidad, populations of guppy (*Poecilia reticulata*) exist in a wide range of polluted environments, and they could provide a good model for gaining valuable insights into genetic mechanisms involved in local adaptation. *Poecilia reticulata* has a short generation time (females first reproduce at 10–20 weeks [92]). This would allow multi-generational experiments to be conducted that are required to assess fitness costs. Such experiments have shown that *P. reticulata* can adapt to strong selection pressures (e.g. to predation and fishing pressures) over three to four generations [93,94].

*Poecilia reticulata* populations live in rivers in the south of Trinidad that are heavily polluted with crude oil and with typical petrogenic hydrocarbons including numerous toxic PAHs [27]. In some very recent work, we conducted reciprocal transplants between polluted and unpolluted stretches within two southern rivers to investigate adaptation. No differences in survival and growth were found over a 6-day period. However, guppies from an unpolluted river in the north had a small, but significant, reduction in survival and growth in the polluted river [27]. The small difference in survival in that study provided little evidence for adaptation. However, many of the adverse effects of exposure to PAHs occur during early development or manifest during long, continuous exposure (tumours) so would not have been detected in that study.

**Figure 1.**
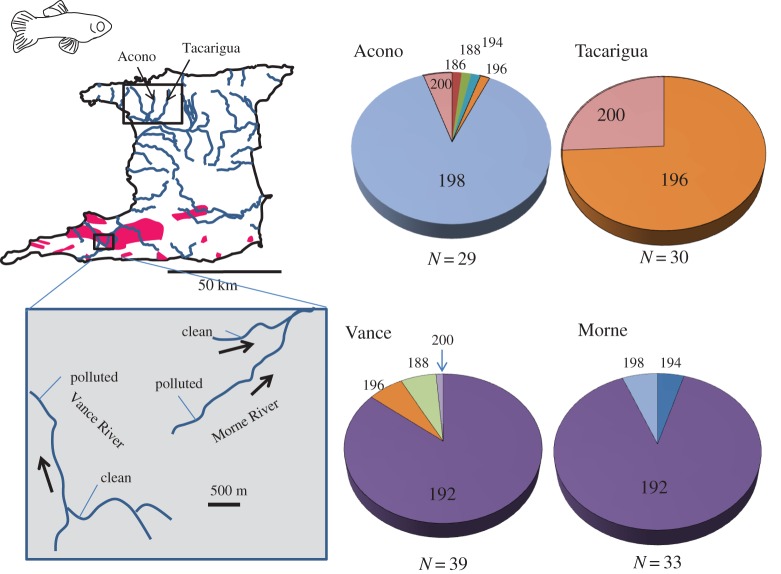
Distribution of alleles for a DNA microsatellite within the CYP1A gene for guppies living in four rivers in Trinidad. The Vance and Morne rivers are unconnected by freshwater and have stretches that are heavily contaminated with crude oil, described in [27]. Within each polluted river, data are pooled for populations from unpolluted and polluted stretches as no frequency differences were observed. Numbers within the pie charts are the sizes (bp) of each allele.

The evolution of tolerance in several populations of *F. heteroclitus* exposed to PAHs has involved altered metabolism by CYP1A (see §3b), and this has been linked to selection at this locus [8]. To investigate evidence for genetic adaptation in these *P. reticulata* populations, we sequenced an exon of CYP1A gene in eight guppies from a highly polluted stretch of the Vance River and nine from a comparatively unpolluted stretch of the Tacarigua River in the north of the country to examine population differentiation. No common alleles were observed between the populations, with fixed SNP differences (*F*_ST_ of 1) between the populations (electronic supplementary material, figure S1). RAD-seq genotyping of eight guppies from each location revealed a mean *F*_ST_ of 0.17 derived from 30 423 polymorphic RAD loci. Only 429 of these RAD loci (1.4%) had an *F*_ST_ of 1, equivalent to that of CYP1A, suggesting that selection had occurred at the CYP1A locus. Further examination of variation by genotyping a DNA microsatellite within the CYP1A locus revealed a high frequency of a single allele (192) in populations from both polluted rivers that was absent from two populations from the north (figure 1). Populations from unpolluted stretches of the polluted rivers also had a high frequency of allele 192, therefore providing no evidence of selection at this gene within the polluted rivers. Of the 1623 RAD-seq loci showing large frequency differences (*F*_ST_ > 0.75, *p* ≤ 0.000001) between the northern and southern populations, 591 fell within genes that were significantly enriched for gene ontology (GO) terms (after Benjamini–Hochberg correction): biological adhesion, cellular process, extracellular matrix and metal ion binding (see the electronic supplementary material for gene lists). Our data did not provide any information on selection at the AHR gene which regulates biological responses to PAH exposure, although it regulates cell adhesion and matrix metabolism which were enriched in this study [95]. The enriched metal ion binding GO term could relate to adaptations to metal contamination. However, among the 59 genes in this group were genes for androgen receptor, superoxide dismutase 1, soluble transferrin-a and Rho-class glutathione S-transferase indicating some of the processes considered in this paper, including metabolism and oxidative stress response, could be involved in adaptation to oil pollution. More extensive analyses with more fish from a greater number of locations are required to control for regional differences unrelated to oil exposure.

## 5. Future directions and knowledge gaps

Knowledge of adaptation will increase in the near future through the application of genetic methods to identify regions of the genome that have been subject to selection. Understanding the importance of local adaptation to contaminant exposure will require studies on a wide range of fish species with different ecological characteristics and that live in habitats with different pollutant profiles. A major challenge will be how to use this information to better manage populations of wild fish. It should also be recognized that adaptation will not necessarily rescue populations [14]. For example, even if the potential to adapt exists, other species with higher tolerance to polluted conditions could out-compete more vulnerable species before adaptations have time to develop.

There are a number of fisheries management practices that could benefit from improved knowledge of adaptation to pollution. For instance, there is increasing interest in improving connectivity in river systems by installing fish passes or removing weirs in order to improve in-river migration and access to spawning grounds. Increased gene flow could disrupt locally adapted populations (maladaptive gene flow), but on the other hand could enable resistant genotypes to spread between populations.

For certain species of fish, it has become common practice to restock rivers after local fish kills. Knowledge of adaptation could influence the decision of whether to allow natural recolonization or to restock with hatchery fish. The use of fish that are genetically resistant to pollution could improve the success of restocking polluted habitats. Likewise, if adaptation has fitness costs, the use of fish that have not adapted to pollutant exposure could improve the success of restocking unpolluted habitats. Additionally, translocation of fish from unpolluted habitats after water quality improvements would aid the establishment of healthy populations. The presence of healthy fish populations (in terms of numbers) in polluted environments could nevertheless adversely impact on other organisms by contaminant transfer to their predators.

## Supplementary Material

Table of microsatellite genotypes

## Supplementary Material

Methods, Gene Ontology tables and Sequence Alignment

## Data accessibility

Raw RAD-seq data are available under study accession PRJEB14708. Sample accessions are ERS1237850-ERS1237865. Accession numbers for CYP1A sequences from the Vance are KX579488 and the Tacarigua KX579489.
