# Supplementary material for: Adaptive capabilities and fitness consequences associated with pollution exposure in fish
Source: Philos Trans R Soc Lond B Biol Sci. 2017 Jan 19;372(1712):20160042. doi: 10.1098/rstb.2016.0042 (PMC5182438; doi:10.1098/rstb.2016.0042)
Supplement: Methods, Gene Ontology tables and Sequence Alignment [file rstb20160042supp2.pdf]

Supplementary information

Title

Adaptive Capabilities and Fitness Consequences Associated with Pollution Exposure in Fish

Authors

Patrick B. Hamilton[1], Gregor Rolshausen[2], Tamsyn M. Uren Webster[3], Charles R. Tyler[1]

[1] University of Exeter, Biosciences, College of Life and Environmental Sciences, Exeter, EX4 4QD, UK

[2] Senckenberg Biodiversity and Climate Research Centre (BiK-F), Senckenberganlage 25, 60325 Frankfurt am Main, Germany

[3] Swansea University, Wallace Building, Swansea, SA2 8PP, UK

Methods

Sampling locations

Details of sampling locations in the Vance and Morne rivers are described in [1]. Within the Vance, 20 fish were sampled from the polluted stretch and 19 from the clean and within the Morne 17 were sampled from the polluted stretch and 16 from the clean. 30 fish were sampled from a relatively clean location in the Tacarigua River is N10^{o}41’229’’ W061 21’31.5 and 29 fish were sampled from a relatively clean stretch of the Acono River is N10^{o}42’484’’ W061 23’52.5.

Cyp1A genotyping

Microsatellite loci were amplified using the Type-it Microsatellite PCR Kit (Qiagen) with a total volume of 9 µl. Primers used for amplification were Cyp1A_ms_f (ACCATTCCATTCAAGTTTGTAAGC) and Cyp1A_ms_4 (TCATGTTTTGGCACTGACAAG) corresponding to positions 2113 -2136 and 2312-2292 in XM_008405514 respectively. Amplicons were run on a Beckman Coulter DNA sequencer. Microsatellite genotypes were determined using the Fragment Analysis on CEQ 8000 (Beckman Coulter). A ‘touchdown’ PCR program was used, consisting of 5 min at 95 ^{o}C followed by 35 cycles of 30 s at 95 ^{o}C, 30 s at the annealing temperature [62 ^{o}C (2 cycles), 58 (3), 55 (5), 53 (10), 51 (5), 49 (5), 47 (5)] and 1 min at 72, then 72 and 60 ^{o}C for 10 and 35 min respectively.

The sequences of primers used for amplification and sequencing the 718 bp fragment of the Cyp1A gene (206-932 bp in XM_008405514.1) were forward (CTWCGTCRACTCCCYGGCCC) and reverse (CAAARHGTAGTGCTCRSTGAC). Phusion® High-Fidelity DNA Polymerase (NEB) was used for PCR amplification in order to minimise PCR errors. PCR products were run on an agarose gel, purified and then sent for Sanger sequencing (Source Biosciences, Nottingham). Consensus sequences were assembled from electropherogram alignments using DNABASER (http://www.dnabaser.com/home.html).

Restriction site associated (RAD)-seq genotyping

Restriction site associated RAD libraries were prepared according to the methodology described in Etter et al. [2]. Briefly, 1.0 µg DNA per from each fish sample was digested with *Sbf*I-HF (NEB). Digested DNA was ligated to P1 adaptors with barcodes unique for each fish (Table S1). These 16 samples were pooled with other samples from another project for paired-end sequencing (100 × 2 bp target) on a single lane of an Illumina HiSeq 2500 at the University of Exeter Sequencing Service. Summary statistics of raw sequencing reads are given in Table S1.

Table S1 - Summary statistics of raw sequencing reads, and mean merged stack depth coverage for guppies analysed in this project.

Sample code	River	Barcode	Total sequence reads with barcode	No. sequences retained	Merged Stack depth coverage
GupVan1	Vance	GAAGC	14576202	13230669	98.5
GupVan2	Vance	GACTA	11869056	10831399	90.2
GupVan3	Vance	GAGAT	16509604	14849446	114.3
GupVan4	Vance	GATCG	7492410	6805138	64.5
GupVan5	Vance	GCATT	6181344	5394675	42.3
GupVan6	Vance	GCCGG	6474992	5880635	48.6
GupVan7	Vance	GCGCC	3026428	2653012	25.3
GupVan8	Vance	GCTAA	4876894	4376323	39.3
GupTacCl1	Tacarigua	GGAAG	7396370	6672907	56.7
GupTacCl2	Tacarigua	GGCCT	8179000	7388879	60.4
GupTacCl3	Tacarigua	GGGGA	9441248	8562389	68.8
GupTacCl4	Tacarigua	GGTTC	7649536	6907453	53.7
GupTacCl5	Tacarigua	GTACA	8164260	7474067	59.5
GupTacCl6	Tacarigua	GTCAC	5854056	5283060	41.0
GupTacCl7	Tacarigua	GTGTG	3559848	3082145	28.9
GupTacCl8	Tacarigua	GTTGT	8081314	7314693	60.3

We used Stacks 1.29 [3-4] for SNP discovery and genotyping from the sequenced RAD tags. ‘process_radtags’ was used for quality filtering of raw reads and demultiplexing based on barcodes, ‘ustacks’ was used to assemble stacks of similar sequences in each individual and a catalog of loci was created with ‘cstacks’. Parameter values used were: minimum depth of coverage required to create a stack (m) = 3, maximum distance (in nucleotides) allowed between stacks (M) = 3, and maximum distance allowed to align secondary reads to primary stacks (n) = 1. FST values were calculated in ‘populations’ using SNPs that were retained in more than 80% of individuals.

We examined for enriched gene ontology (GO) terms in RAD loci which had FST values of greater than 0.75. This stringent cutoff was used because of the low number of fish sampled from each river (n=8). BLASTx [5] against the zebrafish Ensembl [6] peptide database was used to identify RAD loci that fell within genes, using an *e* value cut off of $< 1 \times 10^{-5}$. Of the 1632 loci with FST > 0.75, 591 fell within 493 unique genes. GO analysis was conducted in Database for Annotation, Visualisation and Integrated Discovery (DAVID) [7-8], using the *Danio rerio* as a background. Overrepresented GO terms are shown in Table S2, and Tables S3-S7 list the overrepresented genes within these categories.

Table S2 - Gene Ontology Terms over-represented in the list of genes with an FST > 0.75 between guppy populations from the oil-polluted Vance River and the relatively clean Tacarigua River. This analysis was conducted using the Database for Annotation, Visualisation and Integrated Discovery (DAVID) [7-8], using the *Danio rerio* as a background. Only terms that were significant ($p < 0.05$) after Benjamini−Hochberg correction are shown.

**Category**	**Term**	**Count**	**%**	**P-value**	**Benjamini**
GOTERM_BP_1	biological adhesion	10	2.9	3.40E-03	5.70E-02
GOTERM_BP_1	cellular process	83	23.7	7.10E-03	5.80E-02
GOTERM_CC_2	extracellular matrix part	4	1.1	3.80E-03	9.30E-02
GOTERM_MF_2	ion binding	59	16.9	8.60E-05	2.70E-03
GOTERM_MF_3	cation binding	59	16.9	2.80E-04	1.50E-02
GOTERM_MF_4	metal ion binding	58	16.6	4.70E-04	2.50E-02
GOTERM_MF_4	glycosaminoglycan binding	4	1.1	2.00E-03	5.10E-02

Table S3 - Genes within overrepresented GO term ‘biological adhesion’ (GOTERM_BP_1).

ENSEMBL_GENE_ID	GENE NAME
ENSDARG00000058543	laminin, alpha 5
ENSDARG00000039133	laminin, beta 4
ENSDARG00000052494	protocadherin 18b
ENSDARG00000018542	si:dkey-19f23.2
ENSDARG00000045748	si:dkey-207j16.6
ENSDARG00000009123	similar to P-selectin precursor (Granule membrane protein 140) (GMP-140) (PADGEM) (Leukocyte-endothelial cell adhesion molecule 3) (LECAM3) (CD62P antigen); si:ch211-260g14.8
ENSDARG00000071865	similar to neuropilin-1; neuropilin 1a
ENSDARG00000076484	stabilin 1-like
ENSDARG00000010785	thrombospondin 1
ENSDARG00000077641	thrombospondin 3a

Table S4 - Genes within overrepresented GO term ‘cellular process’ (GOTERM_BP_1).

ENSEMBL_GENE_ID	GENE NAME
ENSDARG00000019404	ATP synthase, H+ transporting, mitochondrial F1 complex, delta subunit
ENSDARG00000058953	ATP-binding cassette, sub-family C (CFTR/MRP), member 4
ENSDARG00000076833	ATPase, Na+/K+ transporting, beta 1b polypeptide
ENSDARG00000062521	ATPase, class II, type 9B
ENSDARG00000069857	F-box and leucine-rich repeat protein 4; coenzyme Q3 homolog, methyltransferase (yeast)
ENSDARG00000076856	Fras1 related extracellular matrix protein 2a
ENSDARG00000001313	G2/M-phase specific E3 ubiquitin ligase
ENSDARG00000017366	PR domain containing 4
ENSDARG00000058306	PRP18 pre-mRNA processing factor 18 homolog (yeast)
ENSDARG00000017397	SWI/SNF related, matrix associated, actin dependent regulator of chromatin, subfamily c, member 1
ENSDARG00000063708	TANK-binding kinase 1
ENSDARG00000006283	Wiskott-Aldrich syndrome-like b
ENSDARG00000013058	activating signal cointegrator 1 complex subunit 2
ENSDARG00000067976	androgen receptor
ENSDARG00000060687	chromodomain helicase DNA binding protein 2
ENSDARG00000034763	cyclin F

ENSDARG00000030053	elongation factor-1, delta, b
ENSDARG00000019489	general transcription factor IIB
ENSDARG00000056642	histone deacetylase 9b
ENSDARG00000061665	hyperpolarization activated cyclic nucleotide-gated potassium channel 2
ENSDARG00000021389	jagged 2
ENSDARG00000062053	kif1-binding protein
ENSDARG00000012073	kinesin family member 15
ENSDARG00000058543	laminin, alpha 5
ENSDARG00000039133	laminin, beta 4
ENSDARG00000006029	leukotriene A4 hydrolase
ENSDARG00000028559	membrane-associated ring finger (C3HC4) 5, like
ENSDARG00000033440	methionyl aminopeptidase 1
ENSDARG00000004537	myeloid/lymphoid or mixed-lineage leukemia (trithorax homolog, Drosophila)
ENSDARG00000033757	non-SMC condensin II complex, subunit H2
ENSDARG00000075887	novel protein similar to vertebrate hect domain and RLD 3 (HERC3)
ENSDARG00000059604	novel protein similar to vertebrate metastasis suppressor 1 (MTSS1)
ENSDARG00000036546	novel protein similar to vertebrate pyridoxal (pyridoxine, vitamin B6) kinase (PDXK); pyridoxal (pyridoxine, vitamin B6) kinase
ENSDARG00000077404	nuclear receptor coactivator 3
ENSDARG00000035095	one-eyed pinhead
ENSDARG00000055026	patched 1
ENSDARG00000013522	phosphoenolpyruvate carboxykinase 1 (soluble)
ENSDARG00000057562	polymerase (RNA) III (DNA directed) polypeptide C
ENSDARG00000031907	polypyrimidine tract binding protein 1b
ENSDARG00000013968	prosaposin
ENSDARG00000052494	protocadherin 18b
ENSDARG00000017369	semaphorin 3d
ENSDARG00000017437	si:ch211-153j24.5
ENSDARG00000045880	si:ch211-203h15.1
ENSDARG00000059707	si:ch211-216l23.1
ENSDARG00000062402	si:ch211-219p7.1; Fras-related extracellular matrix protein 1b
ENSDARG00000039392	si:ch211-240l19.1
ENSDARG00000063535	si:ch211-51m24.3
ENSDARG00000018542	si:dkey-19f23.2
ENSDARG00000045748	si:dkey-207j16.6

ENSDARG00000009123	similar to P-selectin precursor (Granule membrane protein 140) (GMP-140) (PADGEM) (Leukocyte-endothelial cell adhesion molecule 3) (LECAM3) (CD62P antigen); si:ch211-260g14.8
ENSDARG00000002385	similar to Serine-protein kinase ATM (Ataxia telangiectasia mutated) (A-T, mutated); ataxia telangiectasia mutated
ENSDARG00000005456	similar to katanin p80 (WD repeat containing) subunit B 1; katanin p80 (WD repeat containing) subunit B 1
ENSDARG00000071865	similar to neuropilin-1; neuropilin 1a
ENSDARG00000013855	solute carrier family 12 (sodium/chloride transporters), member 3
ENSDARG00000007180	solute carrier family 30 (zinc transporter), member 4
ENSDARG00000000241	solute carrier family 40 (iron-regulated transporter), member 1
ENSDARG00000019231	spectrin alpha 2
ENSDARG00000076484	stabilin 1-like
ENSDARG00000043848	superoxide dismutase 1, soluble
ENSDARG00000010785	thrombospondin 1
ENSDARG00000077641	thrombospondin 3a
ENSDARG00000028213	titin a
ENSDARG00000016771	transferrin-a; Rho-class glutathione S-transferase
ENSDARG00000071586	transforming growth factor, beta-induced
ENSDARG00000071197	ubiquitin specific peptidase 40
ENSDARG00000037017	ubiquitination factor E4B, UFD2 homolog (S. cerevisiae)
ENSDARG00000006202	v-erb-b2 erythroblastic leukemia viral oncogene homolog 3a
ENSDARG00000079564	ventricular myosin heavy chain
ENSDARG00000058821	wu:fa95e03
ENSDARG00000052139	wu:fc10f03; notch homolog 3
ENSDARG00000037309	zgc:103736; si:ch211-150a22.1
ENSDARG00000044479	zgc:110460
ENSDARG00000052553	zgc:112973
ENSDARG00000014599	zgc:114067
ENSDARG00000061890	zgc:136762
ENSDARG00000063684	zgc:153678
ENSDARG00000007231	zgc:158381
ENSDARG00000055679	zgc:158782
ENSDARG00000061493	zgc:172103
ENSDARG00000015240	zgc:194395
ENSDARG00000016200	zgc:76966
ENSDARG00000042747	zgc:92337; hypothetical LOC792234

Table S5 - Genes within overrepresented GO term ‘extracellular matrix part’ (GOTERM_CC_2).

**ENSEMBL_GENE_ID**	**GENE NAME**
ENSDARG00000035809	collagen, type I, alpha 3
ENSDARG00000058543	laminin, alpha 5
ENSDARG00000039133	laminin, beta 4
ENSDARG00000003395	type IV collagen alpha 3 chain

Table S6 - Genes within overrepresented GO terms ‘ion binding’ (GOTERM_MF_2), ‘cation binding’ (GOTERM_MF_3) and metal ion binding (GOTERM_MF_4). The same genes were in each of these categories, except for galactosidase alpha, which was absent from metal ion binding.

**ENSEMBL_GENE_ID**	**GENE NAME**
ENSDARG00000076833	ATPase, Na+/K+ transporting, beta 1b polypeptide
ENSDARG00000062521	ATPase, class II, type 9B
ENSDARG00000001313	G2/M-phase specific E3 ubiquitin ligase
ENSDARG00000054749	LIM domain only 4, like
ENSDARG00000007429	NADPH dependent diflavin oxidoreductase 1
ENSDARG00000017366	PR domain containing 4
ENSDARG00000013058	activating signal cointegrator 1 complex subunit 2
ENSDARG00000067976	androgen receptor
ENSDARG00000002046	bloodthirsty-related gene family, member 29
ENSDARG00000035329	calpain, small subunit 1 a
ENSDARG00000008219	crystallin, gamma M4; fibulin 4
ENSDARG00000036155	galactosidase, alpha
ENSDARG00000019489	general transcription factor IIB
ENSDARG00000051914	hypothetical LOC554517; zgc:136632
ENSDARG00000027079	hypothetical LOC565294
ENSDARG00000021389	jagged 2
ENSDARG00000006029	leukotriene A4 hydrolase
ENSDARG00000029476	low density lipoprotein receptor
ENSDARG00000028559	membrane-associated ring finger (C3HC4) 5, like
ENSDARG00000033440	methionyl aminopeptidase 1
ENSDARG00000025699	methyl-CpG binding domain protein 1
ENSDARG00000062686	multiple EGF-like-domains 11
ENSDARG00000004537	myeloid/lymphoid or mixed-lineage leukemia (trithorax homolog, Drosophila)
ENSDARG00000029124	novel protein similar to vertebrate ADAM metallopeptidase with thrombospondin type 1 motif, 15 (ADAMTS15)
ENSDARG00000060094	prostaglandin I2 (prostacyclin) synthase like
ENSDARG00000052494	protocadherin 18b
ENSDARG00000055416	serpin peptidase inhibitor, clade B (ovalbumin), member 1
ENSDARG00000059707	si:ch211-216l23.1
ENSDARG00000020354	si:ch211-260g14.3
ENSDARG00000063535	si:ch211-51m24.3

ENSDARG00000000161	si:dkey-266k12.1
ENSDARG00000077897	si:dkey-7l12.1
ENSDARG00000009123	similar to P-selectin precursor (Granule membrane protein 140) (GMP-140) (PADGEM) (Leukocyte-endothelial cell adhesion molecule 3) (LECAM3) (CD62P antigen); si:ch211-260g14.8
ENSDARG00000062049	similar to histocompatibility (minor) HA-1; histocompatibility (minor) HA-1
ENSDARG00000054984	similar to zinc finger and BTB domain containing 41
ENSDARG00000000241	solute carrier family 40 (iron-regulated transporter), member 1
ENSDARG00000001549	sp3 transcription factor
ENSDARG00000019231	spectrin alpha 2
ENSDARG00000043848	superoxide dismutase 1, soluble
ENSDARG00000010785	thrombospondin 1
ENSDARG00000077641	thrombospondin 3a
ENSDARG00000016771	transferrin-a; Rho-class glutathione S-transferase
ENSDARG00000005397	tripartite motif-containing 3b
ENSDARG00000052139	wu:fc10f03; notch homolog 3
ENSDARG00000043016	zgc:112020
ENSDARG00000052553	zgc:112973
ENSDARG00000058830	zgc:154029
ENSDARG00000058842	zgc:162740
ENSDARG00000012184	zgc:162872
ENSDARG00000070156	zgc:162971
ENSDARG00000075733	zgc:171680
ENSDARG00000061493	zgc:172103
ENSDARG00000036929	zgc:175173
ENSDARG00000057550	zgc:193699; similar to Tripartite motif-containing protein 62
ENSDARG00000015222	zgc:55308
ENSDARG00000021859	zgc:56194
ENSDARG00000027182	zgc:66260
ENSDARG00000026453	zgc:66474
ENSDARG00000027887	zinc finger, FYVE domain containing 21

Table S7 - Genes within overrepresented GO term ‘glycosaminoglycan binding’ (GOTERM_MF_4).

**ENSEMBL_GENE_ID**	**GENE NAME**
ENSDARG00000018542	si:dkey-19f23.2
ENSDARG00000045748	si:dkey-207j16.6
ENSDARG00000076484	stabilin 1-like
ENSDARG00000077641	thrombospondin 3a

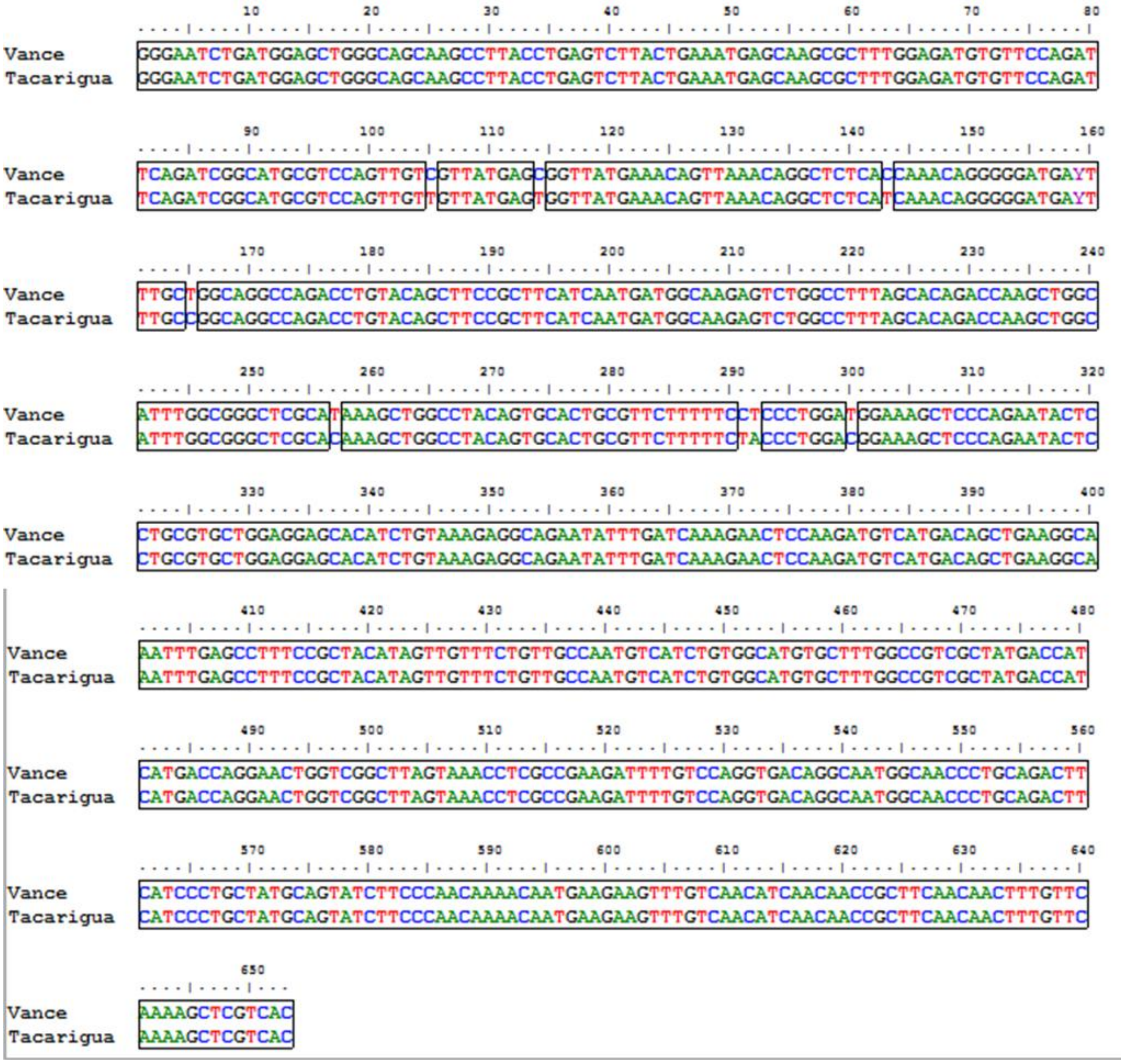

Figure S1 - Alignment of consensus Cyp1A gene sequences from the guppies originating from the oil-polluted Vance River and the comparatively clean Tacarigua River highlighting fixed SNP differences between the populations.

References

[1] Rolshausen, G., Phillip, D.A.T., Beckles, D.M., Akbari, A., Ghoshal, S., Hamilton, P.B., Tyler, C.R., Scarlett, A.G., Ramnarine, I., Bentzen, P., et al. 2015 Do stressful conditions make adaptation difficult? Guppies in the oil-polluted environments of southern Trinidad. *Evol. Appl.* **8**, 854–870. (doi:10.1111/eva.12289).

[2] Etter, P., Bassham, S., Hohenlohe, P., Johnson, E. & Cresko, W. 2011 SNP Discovery and Genotyping for Evolutionary Genetics Using RAD Sequencing. In *Molecular Methods for Evolutionary Genetics* (eds. V. Orgogozo & M.V. Rockman), pp. 157-178, Humana Press.
[3] Catchen, J., Hohenlohe, P.A., Bassham, S., Amores, A. & Cresko, W.A. 2013 Stacks: an analysis tool set for population genomics. *Mol. Ecol.* **22**, 3124-3140. (doi:10.1111/mec.12354).
[4] Catchen, J.M., Amores, A., Hohenlohe, P., Cresko, W. & Postlethwait, J.H. 2011 Stacks: building and genotyping loci de novo from short-read sequences. *G3: Genes, Genomes, Genetics* **1**, 171-182. (doi:10.1534/g3.111.000240).
[5] Altschul, S.F., Gish, W., Miller, W., Myers, E.W. & Lipman, D.J. 1990 Basic local alignment search tool. *J. Mol. Biol.* **215**, 403-410. (doi:10.1006/jmbi.1990.9999).
[6] Flicek, P., Amode, M.R., Barrell, D., Beal, K., Billis, K., Brent, S., Carvalho-Silva, D., Clapham, P., Coates, G., Fitzgerald, S., et al. 2014 Ensembl 2014. *Nucleic Acids Res.* **42**, D749-D755. (doi:10.1093/nar/gkt1196).
[7] Huang, D.W., Sherman, B.T. & Lempicki, R.A. 2008 Systematic and integrative analysis of large gene lists using DAVID bioinformatics resources. *Nat. Protoc.* **4**, 44-57. (doi:http://www.nature.com/nprot/journal/v4/n1/suppinfo/nprot.2008.211_S1.html).
[8] Huang, D.W., Sherman, B.T. & Lempicki, R.A. 2009 Systematic and integrative analysis of large gene lists using DAVID bioinformatics resources. *Nat. Protoc.* **4**, 44-57. (doi:10.1038/nprot.2008.211).
